# The knowledge, attitudes and practices of wintersun vacationers to the Gambia toward prevention of malaria: is it really that bad?

**DOI:** 10.1186/1475-2875-13-74

**Published:** 2014-02-28

**Authors:** Perry JJ van Genderen, Paul GH Mulder, David Overbosch

**Affiliations:** 1 Institute for Tropical Diseases, Havenziekenhuis, Haringvliet 72, 3011 TG Rotterdam, The Netherlands; 2 Travel Clinic Havenziekenhuis, Rotterdam, The Netherlands; 3 Department of Biostatistics, Erasmus University Hospital, Rotterdam, The Netherlands

**Keywords:** Malaria, Traveller, Travel, Risk, Knowledge, Attitude, Practice, KAP, VFR, Gambia, Tourist

## Abstract

**Background:**

Each year clusters of imported malaria cases are observed in Dutch wintersun vacationers returning from The Gambia. To gain more insight in the travel health preparation and awareness of these travellers, the knowledge, attitudes and practices (KAP) of this travel group was studied by analysing the data of the Continuous Dutch Schiphol Airport Survey.

**Methods:**

In the years 2002 to 2009 a questionnaire-based survey was conducted at the Dutch Schiphol Airport with the aim to study the KAP, i.e. accuracy of risk perception (“knowledge”), intended risk-avoiding behaviour (“attitude”) and use of personal protective measures and malaria chemoprophylaxis (“practice”) toward prevention malaria in travellers to The Gambia. Travellers to other high-risk destinations served as controls.

**Results:**

The KAP of travellers to The Gambia toward prevention of malaria was significantly better than that observed in other travellers. Trend analyses indicated that attitude improved over time in both groups but knowledge did not change. Only in travellers to high-risk countries other than The Gambia significant increases in protection rates were observed over time.

**Conclusions:**

The KAP of travellers to The Gambia toward prevention of malaria was better than that observed in travellers to destinations other than The Gambia. Trend analyses revealed a significant improvement of intended risk avoiding behaviour but not in protection rates or risk perception.

## Background

In the Netherlands, vacations to The Gambia are frequently marketed as attractive last-minute ‘winter sun’ alternatives for the Canary Islands, Portugal or Spain. However, being located in West-Africa, travel to The Gambia requires not only proof of protection against yellow fever but has also strict indications for malaria chemoprophylaxis throughout the year. However, many travel brochures and booking agencies underexpose the need for malaria prophylaxis and proper travel health advice [1]. As a consequence, travellers to The Gambia are considered to be at an increased risk for contracting malaria because of this lack of awareness and prophylactic measures. In fact, clusters of imported malaria cases in wintersun vacationers returning from the Gambia were described in several European countries, including the Netherlands [2-4]. Last-minute booking, not seeking or adhering to travel health advice and not taking any or using inappropriate malaria chemoprophylaxis as well as a high case-fatality rate were the common denominators among these cases [2-4], stressing the need for proper preventive measures and increased awareness of the potential life-threatening dangers associated with travel to West Africa for this group of travellers.

In an effort to gain more insight in the travel health preparation and awareness of particularly wintersun vacationers to the Gambia, the knowledge, attitudes and practices (KAP) of this travel group was studied by analysing the data of the Continuous Dutch Schiphol Airport Survey. In this annually repeated questionnaire-based survey, the main determinants that constitute the traveller’s personal risk profile toward travel-related infectious diseases like malaria were systematically evaluated in passengers waiting to board on flights to various destinations with a risk for contracting malaria and provide important feed-back on their travel health preparation, perception of risk as well as risk-seeking or risk-avoiding behaviour.

## Methods

### Questionnaires and survey

The survey was conducted as previously described [5-8]. However, for the current study only travellers to a destination with a designated high risk of malaria were included based on maps published by the Center for Disease Control, Atlanta, USA [9]. In brief, self-administered, anonymous questionnaires were randomly distributed at the departure gate of Schiphol Airport, Amsterdam, The Netherlands, while passengers were waiting to board. Intercontinental flights to destinations with an intermediate or high risk for hepatitis A, hepatitis B or malaria were preferably selected. The survey was always done in the same period of the year, namely the months October or November in the years 2002 to 2009, except in year 2006. Travellers participated on a voluntary basis; no incentive was provided, except for a leaflet with information on hepatitis A, hepatitis B and malaria. Trained interviewers were present to distribute the questionnaires, to answer questions if necessary and to check the completeness of the responses collected. When possible, these interviewers copied the information from the travellers’ vaccination records. Travellers were allowed to participate if they were 18 years of age or older, and able to fully understand the language of the questionnaires. They also had to be resident in the Netherlands; thus, nationals of a developing country were only asked to participate if they were actually living in the Netherlands. These criteria were checked by the interviewers when distributing the forms. Afterwards, completed questionnaires from travellers who did not meet all the inclusion criteria were either excluded by the interviewers or rejected from the final analysis.

The malaria questionnaire focused on malaria and its prevention and treatment and these questionnaires were distributed only to travellers with destinations in or close to malaria-endemic areas. A part of the questionnaire dealt with personal characteristics (age, gender, nationality, residence, profession), with information regarding the travel (destination, duration, purpose, travel companions) and its preparation, and with the travellers’ intended risk-seeking behaviour as well as perception of risk of malaria at their destination.

### Determination of KAP profile on malaria

Knowledge of malaria was determined by comparison of the risk for malaria as perceived by the traveller with the actual risk for malaria, as described [8]. For the current study, only destinations with a known high risk for malaria based on maps published by the Center for Disease Control, Atlanta, USA [9] were included. For each subject the accuracy (correct risk perception) was expressed as 0 or 1, with 1 assigned to a subject if his (her) knowledge about risk was compatible with the official risk rating of the destination. To determine the attitude (intended risk taking or risk avoiding behaviour) of participants towards prevention of malaria, all participants were asked if they were planning to: (1.) cover their arms and legs when going outside; (2.) use of an insect repellent on uncovered skin; (3.) keep the doors and windows closed; (4.) sleep under a bed net and (5.) stay in air-conditioned surroundings. Each affirmative answer was scored with 1 point whereas a negation was scored with 0 points. The final attitude score towards prevention of malaria was obtained as the sum of the separate answer scores and could, therefore, range from 0 to 5; for convenience, the score was transformed to a 0–100 scale with the maximal protective attitude score set at 100. To have an indication of their practice (protection rate) towards prevention of malaria travellers were asked whether they had packed personal protective measures like insect repellents, bed nets and malaria chemoprophylaxis for this trip. Protection rate was expressed as a weighted sum of use of insect repellent (1 point), use of bed net (2 points) and use of malaria chemoprophylaxis (3 points). The practice sum score could, therefore, range from 0 to 6; for convenience, the score was transformed to a 0–100 scale with the maximal practice score set at 100. In order to estimate the impact of KAP of the travel risk group of interest on relative risk for malaria, a composite risk estimate was constructed by summing up the effects of the separate determinants. To that end, it was assumed that either a poor risk perception, intended risk-seeking behaviour or poor protection rates led to an equal increase in relative risk for malaria.

### Statistical analysis

Travellers to The Gambia were compared with travellers to other destinations carrying a high risk for acquiring malaria. Differences in general characteristics between The Gambia and other high malaria risk destinations were tested using multiple logistic regression analyses, adjusted for survey year (7 nominal categories). Each of the risk factors knowledge, attitude score and practice score was analysed as dependent variable in a multiple regression analysis with destination (The Gambia versus other high risk destination) and survey year as categorical independent variables. Alternatively, survey year was entered in the regression models as a numeric trend variable along with its interaction term with destination (The Gambia versus other high risk destination). For the dichotomous dependent variable knowledge a multiple logistic regression model was used; for the attitude and practice scores multiple linear regression models were used. A *P*-value below 0.05 was considered to denote statistical significance. The estimated effect and its 95% confidence interval are presented for a *P*-value below 0.10.

## Results

### Study population

Across all seven years in the period from 2002 to 2009 (except year 2006) there were 3,045 eligible respondents in the Dutch Continuous Schiphol Airport study, as previously published [8]; 708 of them travelled to destinations with a high risk for malaria and were included in the current study. 373 (52.7%) of them travelled to The Gambia. 335 respondents travelled to other high-risk destinations, most commonly to Surinam (n = 101, 14.3%) followed by Ghana (n = 66, 9.3%), Nigeria (n = 49, 6.9%), Uganda (n = 31, 4.4%) and Kenya (n = 29, 4.1%), respectively.

The general characteristics of the 708 travellers, grouped by destination to The Gambia or to other high-risk destinations, are shown in Table 1. Overall, 49.4% of responders were female and 50.6% were male. Almost 71% of the travellers used malaria chemoprophylaxis and had packed insect repellents.

**Table 1:** General characteristics of 708 respondents traveling to a destination with a high risk of malaria

	**Gambia**		**Other high risk destinations**		**P-value**^**2**^
	**N**	**%**	**N**	**%**	
	**373**	**52.7**	**335**	**47.3**	
Sex					
*Male*	182	48.8^1^	176	52.5^1^	n.s.
*Female*	186	49.9	159	47.5	
Age					
*Age > 60 yrs*	79	21.2	49	14.6	n.s.
Travel duration					
*< 7 days*	80	21.4	59	17.6	<0.001
*8 - 14 days*	165	44.2	77	23.0	
*15 - 28 days*	80	21.4	87	26.0	
*> 28 days*	18	4.8	69	20.6	
Travel health preparation					
Pre-travel information					n.s.
*No*	39	10.5	69	20.6	
*Yes*	334	89.5	266	79.4	
Information source					0.037
*Travel clinic/publich health service*	216	57.9	145	43.3	
*Company doctor*	4	1.1	13	3.9	
*General practitioner/pharmacy*	43	11.5	29	8.7	
*Other*	17	4.6	23	6.9	
Time frame information - departure					n.s.
*< 7 days*	45	12.1	34	10.1	
*8 - 14 days*	66	17.7	49	14.6	
*15 - 28 days*	83	22.3	76	22.7	
*> 28 days*	140	37.5	107	31.9	
Purpose for travel					<0.001
*Tourist*	271	72.7	110	32.8	
*Business*	26	7.0	76	22.7	
*VFR*	46	12.3	108	32.2	
*Missionary/volunteer*	16	4.3	27	8.1	
*Research*	6	1.6	7	2.1	
*Other*	5	1.3	5	1.5	
Travel profile					<0.001
*Solo traveller*	69	18.5	140	41.8	
*Travel with spouse*	209	56.0	77	23.0	
*Travel with children*	32	8.6	36	10.7	
*Travel with group*	19	5.1	28	8.4	
*Travel with friends*	26	7.0	17	5.1	
*Other*	11	2.9	18	5.4	
Preventive measures					
*Insectrepellents*	323	86.6	178	53.1	<0.001
*Bednet*	74	19.8	64	19.1	0.027
*Chemoprophylaxis*	323	86.6	175	52.2	<0.001

^1^all data are given as a percentage of either the total number of respondents travelling to The Gambia (i.e., n = 373) or as a percentage of the total number of respondents travelling to an other high risk destination (i.e., n = 335). Values do not always add up to 100% due to missing values; ^2^P-value for comparison of travellers to The Gambia *vs* other high risk destinations, adjusted for year of survey through multiple logistic regression analysis.

### Travel profile

For 20.3% of the travellers since 2004 it was their first trip to a developing country (there was no first-trip-item in the questionnaires of 2002 and 2003). This percentage did not significantly differ between travellers to The Gambia and those to other high-risk destinations (*P* = 0.44), adjusted for survey year. Overall, 53.8% indicated tourism as their purpose of travel, 42.6% were visiting friends and relatives, business travellers accounted for 14.4%. Travellers to The Gambia were most often tourist whereas VFRs comprised a substantial proportion of the travellers to other high-risk destinations. The travel profile also significantly differed: many travellers to The Gambia (56.0%) were accompanied by their partner or spouse, whereas a substantial proportion (41.8%) of the travellers to other high-risk destinations travelled alone. Travellers to other high-risk destinations planned to stay significantly longer on their destination than travellers to the Gambia (p < 0.001), but did not obtain pre-travel health advice more frequently prior to departure (p < 0.001).

### Travel health preparations

The majority of travellers (84.7%) had sought health information about their destination prior to departure. Travellers to The Gambia did not differ from travellers to other high-risk destinations with regard to whether or not seeking health information or timing of collection of information. The most common sources since 2004 for travel health advice to high-risk destinations were the travel clinic or public health service (27.8%) followed by general practitioner (GP). In the 2002- and 2003-questionnaires there was no item concerning source of advice. There was a significant positive trend over the years in the proportion of travellers to The Gambia seeking travel health advice: odds ratio of 1.23 per year (95% CI: 1.05-1.45; P = 0.012). For travellers to other high-risk destinations this trend was not significant: odds ratio of 1.11 per year (95% CI: 0.99-1,23; P = 0.067).

### Knowledge, attitudes and practices toward prevention of malaria

#### Knowledge (accurate risk perception)

There was no significant difference in knowledge, defined as an accurate risk perception of malaria, between travellers to The Gambia and those to other high-risk destinations, adjusted for survey year (*P* = 0.052); see Table 2. The adjusted knowledge odds ratio of The Gambia relatively to other destinations was 0.67 (95% CI: 0.45-1.00). In addition, no significant time trend in traveller’s knowledge was observed for The Gambia (P = 0.071) or for other destinations (*P* = 0.97), nor did these time trends significantly differ between The Gambia and other destinations (*P* = 0.22).

**Table 2:** Knowledge, attitude and practice of wintersun vacationers to the Gambia in comparison to travellers to other destinations carrying a high risk of transmission of malaria

**Outcome variable**	**Gambia (n = 373)**				**Other high-risk destination (n = 335)**				**Difference**	
	**n**	**Outcome**	**95% CI**	**Time trend**	**n**	**Outcome**	**95% CI**	**Time trend**	**Outcome**	**Time trend**
				**↑↓ *P*-value**				**↑↓ *P*-value**	***P*-value**	***P*-value**
Knowledge *n/N (%)*		273/373 (73.2)	68.4-77.6	↑ 0.071		243/335 (72.5)	67.4-77.3	0.97	0.052	0.22
Attitude *mean (SD)*	328	72.0 (23.3)	79.4-84.6	↑ 0.001	189	71.9 (26.9)	68.0-75.8	↑ 0.001	0.47	0.68
Practice *mean (SD)*	365	65.6 (23.0)	63.2-68.0	0.36	245	56.5 (32.9)	52.3-60.7	↑ <0.0005	0.001	0.046

*P*-values were calculated after adjustment for year of survey; of other parameters the unadjusted estimates are presented.

#### Attitude (intended risk-avoiding behaviour)

There were no significant difference in attitude between travellers to The Gambia and travellers to other high-risk destinations (*P* = 0.47), adjusted for survey year; see Table 2. In both groups, however, a significant trend toward a risk-avoiding attitude could be established over the years (both *P*-values 0.001). Among travellers to The Gambia risk-avoiding attitude improved by 2.3 points per year (95% CI: 0.91-3.66); among travellers to other high-risk destinations this was by 2.7 points per year (95% CI: 1.14-4.32). These trends did not significantly differ between The Gambia and other high-risk destinations (*P* = 0.68).

#### Practice (protection rate)

Travellers to The Gambia had a 7.6 points higher protection rate than travellers to other high-risk destinations (95% CI: 3.01-12.16; *P* = 0.001), adjusted for survey year; see Table 2. However, only for travellers to other high-risk destinations the protection rate increased by 2.5 points per year (95% CI: 1.15-3.88; *P* < 0.0005), being a 1.9 points (95% CI: 0.04-3.79; *P* = 0.046) larger increase than the non-significant increment in travellers to The Gambia.

## Discussion

Even though the incidence of imported malaria in The Netherlands and other European countries has declined in the last decade [10,11], each year about 10 of 15,000 Dutch tourists acquire malaria in The Gambia. These malaria cases from The Gambia are not limited to Dutch travellers, but also observed in clusters from other European countries during the winter season [2-4]. Last-minute booking, not seeking or adhering to travel health advice and not taking any or using inappropriate malaria chemoprophylaxis as well as a high case-fatality rate were the common denominators among these cases [2-4]. These wintersun vacationers to The Gambia are considered to be at an increased risk for contracting malaria because of this lack of awareness and prophylactic measures. In the present study detailed information was collected on the knowledge, attitude and practices of travellers to The Gambia with travellers to other high-malaria risk countries as comparators. This study revealed some interesting observations.

First, as compared with travellers to other high-risk malaria countries, a significantly higher proportion of travellers to The Gambia indicated adherence to personal preventive measures like insect repellents, bednets and use of malaria chemoprophylaxis. As a consequence, practices toward prevention of malaria was significantly higher in travellers to The Gambia. In addition, travellers to The Gambia disclosed more (intended) risk-avoiding behaviour than travellers to other high-risk countries whereas risk perception of malaria did not differ between both groups. As a result, the KAP of travellers to The Gambia toward prevention of malaria was significantly higher than that observed in travellers to other high-malaria risk countries. These findings may relate to the observation that the proportion of travellers to The Gambia seeking travel health advice increased significantly over time but not for travellers to other high-risk countries.

Second, since the Schiphol survey was repeated annually, this also allowed for trend analyses. These demonstrated that the overall KAP toward prevention of malaria improved for both travellers to The Gambia and travellers to other high-risk countries, but not for each determinant separately. In particular, the knowledge or accuracy of risk perception did not change in both groups whereas significant increases in protection rates were only observed over time for travellers to high-risk countries other than The Gambia but still below the level observed in travellers to The Gambia. Interestingly, the attitude toward prevention of malaria or intended risk-avoiding behaviour improved over time in both groups.

### Limitations

Questionnaire-based surveys may have some drawbacks which may limit the generalizability of the current findings. For instance, the Dutch Schiphol Airport study was originally designed to study the KAP of travellers to destinations with a high or lower risk for malaria, hepatitis A and hepatitis B and all destinations were selected to meet this requirement. The destinations were not randomly selected from all available risk destinations. As such, a sub-study of the original dataset may have these same limitations as well. Further, the survey was always done in the months October and November of each year, which may have introduced a selection bias since people who travel at this time of year may differ from people who travel during summer vacation in terms of purpose for travel, travel duration and adherence to personal preventive measures. On the other hand, the majority of the reported cases who acquired malaria after travel to The Gambia, contracted the infection in the months October - January. Moreover, one could argue that the traveller’s KAP profile may also be influenced by their prior travel experience. To specifically address this potential confounder, all questionnaires since 2004 contained questions elaborating on this item but no significant differences in prior travel experience were found between travellers to The Gambia or to other high-risk destinations. Finally, one could argue that the acquisition of malaria is not restricted to unprotected travellers alone but may also occur in patients taking malaria chemoprophylaxis. In a recent study [12] it was shown that compliant use of malaria chemoprophylaxis significantly reduced the risks of severe malaria but did not completely abolish the occurrence of infection. Interestingly, the effects of non-compliant use of chemoprophylaxis were virtually indistinguishable from not taking any chemoprophylaxis at all, making a strong plea for strict adherence to chemoprophylactic regimens.

In conclusion, the prospects of the KAP of travellers to The Gambia toward prevention of malaria were more bright than could be envisioned on the current body of literature and certainly better than that of travellers to other destinations with a high malaria risk. Moreover, trend analyses indicated that - over time – the overall KAP toward prevention of malaria is further improving in travellers to The Gambia. Continuous efforts of health care providers to create awareness on the risks associated with travel to malarious regions and on the impact of personal protective measures including chemoprophylaxis are likely to bear more fruit in the future.

## Competing interests

The authors declare that they have no competing interests.

## Authors’ contributions

PJJVG conceived of the study, participated in the data analysis and drafted the manuscript. PGHM participated in the design of the study and performed the statistical analysis. DO conceived of the study, and participated in its design and coordination. All authors read and approved the final manuscript.
